# Synergistic effects of *Pandanus fascicularis* extracts and azithromycin: *in vitro* and *in silico* antimicrobial investigation against MDR clinical strains

**DOI:** 10.3389/frabi.2025.1552382

**Published:** 2025-05-29

**Authors:** Mst. Hajera Khatun, Md Rashedul Islam, Shikha Khatun, Amro Ahmed Zalah, Md. Hadisur Rahman Rony, Mst. Munira Khatun, Emad Essa A. Wasili, Jaytirmoy Barmon, Saad Ahmed Sami, Abdulrahman Hadi Masmali, Ishtiaq Qadri

**Affiliations:** ^1^ Department of Pharmacy, School of Science and Technology, Varendra University, Rajshahi, Bangladesh; ^2^ Department of Biochemistry, Faculty of Science, King Abdulaziz University, Jeddah, Saudi Arabia; ^3^ Advanced Biological Invention Centre (Bioinventics), Rajshahi, Bangladesh; ^4^ King Faisal Medical City for Southern Region, Abha, Saudi Arabia; ^5^ BCSIR Rajshahi Laboratories, Bangladesh Council of Scientific and Industrial Research, Rajshahi, Bangladesh; ^6^ Ethnopharmacology and Computational Phytochemistry Laboratory Bangladesh, Dhaka, Bangladesh; ^7^ Department of Biological Sciences, King Abdulaziz University, Jeddah, Saudi Arabia

**Keywords:** multidrug-resistant bacteria, synergistic effect, clinical strains, antioxidant activity, *Pandanus fascicularis*, natural products

## Abstract

**Background:**

Antibiotic-resistant bacteria are becoming a significant global concern. To combat the spread of resistance or reverse multidrug resistance, developing novel antimicrobials and/or resistance modulators is essential. This study aimed to evaluate the synergistic effects of the methanolic extract of *Pandanus fascicularis* fruits (MEPFF) in combination with azithromycin against multidrug-resistant bacteria.

**Methods:**

Phytochemical analysis along with the determination of total phenolic content (TPC), total flavonoid content (TFC), and total antioxidant capacity (TAC) of MEPFF, was performed using standard procedure. The extract's DPPH free radical scavenging activity was assessed to evaluate its potential antioxidant activity. The minimal inhibitory concentration (MIC) and minimal bactericidal concentration (MBC) of MEPFF against *Staphylococcus aureus*, *Bacillus cereus*, *Escherichia coli*, and *Pseudomonas aeruginosa* were determined, followed by an analysis of the synergistic effect with azithromycin, as well as the activity of azithromycin alone. Subsequently, the drug-likeness, antibacterial activity, and toxicological properties were analyzed using in silico tools.

**Results:**

The quantitative investigation found terpenoids, flavonoids, tannins, phenolics, saponins, cardiac glycosides, and alkaloids in MEPFF. The TFC, TPC, and TAC of MEPFF were found at 183 ± 9.54 mg QE, 248.33 ± 11.06 mg GAE, and 95.33 ± 8.33 mg AAE/gm extract. The extract showed significant antioxidant activity in the DPPH experiment, with an IC50 value of 12.13±0.53 µg/ml. Azithromycin and the extract together have far greater antibacterial action against all four bacterial strains. The minimal inhibitory concentration (MIC) is 3.67 ± 1.15 to 5.83 ± 0.76 mg/mL, while the minimum bactericidal concentration (MBC) is 4.33 ± 1.26 to 7.33 ± 1.04 mg/mL. In silico studies revealed that pandamarilactone-1, nonpandamarilactone-B, and thiamine had the best docking energy (−9.9, −8.9, and −8.5 kcal/mol), suggesting most active compounds against MPh-II protein.

**Conclusion:**

The extract enhances antibiotic therapy and suggests that the aforesaid synergistic drug-herb combinations may treat MDR bacterial infections.

## 1 Introduction

Multidrug-resistant (MDR) microorganisms have emerged rapidly, posing a severe and escalating threat to global public health (Cerceo et al., 2016). The indiscriminate use and overuse of antibiotics have contributed to the evolution of MDR strains, where pathogens alter their targets to evade treatment, making them increasingly difficult to treat (Gaibani et al., 2022). These pathogens are spreading not only among humans but also from humans to animals, exacerbating the public health crisis. With antibiotic-resistant diseases claiming an estimated 70,000 lives each year, the global death toll could rise to a staggering 10 million by 2050, surpassing fatalities related to cancer (Duan et al., 2021). This necessitates the development of novel antimicrobials, including those that work in tandem with antibiotics.

One promising approach is using plant-derived antimicrobials, which are being explored for their potential to work in synergy with traditional antibiotics to combat multidrug-resistant (MDR) strains. Recent studies highlight how plant-based compounds enhance the effectiveness of antibiotics, offering a potential strategy to restore their efficacy (Ramata-Stunda et al., 2022). Additionally, investigating plant chemicals as alternative remedies for resistant strains is becoming increasingly popular, showing encouraging outcomes (Pancu et al., 2021; Ruddaraju et al., 2020; Sibanda and Okoh).

One of the most alarming MDR pathogens is *Staphylococcus aureus*, known for causing skin and soft tissue infections (SSTIs) (Akinduti et al., 2022), and for increasing the risk of serious systemic infections like osteomyelitis, septicemia, and bacteremia (Bouvet et al., 2017; Cupane et al., 2012; Jin et al., 2021). Recent reports indicate that SSTIs are often worsened by *Staphylococcus aureus* biofilm, leading to widespread antibiotic resistance and limiting the effectiveness of available treatments (Deyno et al., 2017). *Bacillus cereus* has been considered to cause food poisoning in humans. It causes gastrointestinal illnesses like diarrhea and emesis (Lewin et al., 2019). It is also responsible for bacteraemia and skin infection in uncontrolled diabetic patients (Esmkhani and Shams, 2022; Orrett, 2000). These pathogens, exhibiting resistance to multiple antibiotic classes, underscore the urgent need for novel antimicrobial solutions (Mills et al., 2022).

Additionally, the rise of multidrug-resistant *Escherichia coli* is increasingly concerning in human and veterinary medicine worldwide. Nearly all vital antimicrobial medications are susceptible to *E. coli*, which often acquires resistance genes primarily through horizontal gene transfer (Arbab et al., 2022; Poirel et al., 2018). Furthermore, *Pseudomonas aeruginosa* is one of the most prevalent bacteria linked to pneumonia and infections acquired during hospital stays (Barbier et al., 2013). It rarely impacts on healthy people, but it has a significant morbidity and fatality rate in patients with cystic fibrosis and other immunocompromised people. The Antibiotic treatment alone or in combination decreases the mortality of severe *P. aeruginosa* infections (Park et al., 2011). Nonetheless, the ability of *P. aeruginosa* to build resistance against various existing antibiotics has made treating its infections highly challenging (Pang et al., 2019).

Natural products play a crucial role in addressing bacterial resistance due to their unique structural diversity and bioactive properties, which may synthetic compounds typically lack (Newman and Cragg, 2016). They offer a wonderful opportunity to uncover new antibacterial agents! This is especially important in light of the growing challenge posed by antibiotic-resistant pathogens, which affect healthcare systems around the world (Von Nussbaum et al., 2006; Wright, 2017). The coastal plant *Pandanus fascicularis* belongs to Pandanaceae family (Baul et al., 2017). The fruits of *Pandanus fascicularis* are known for their distinct flavor and are rich in provitamin A carotenoids when ripe. These vibrant and nutrient-dense fruits are often used to help combat vitamin A deficiency, offering a tasty way to support overall health (Englberger et al., 2003; Fitzgerald, 2003). Additionally, they are a significant source of riboflavin, vitamin C (ascorbic acid), niacin (vitamin B3) and thiamine (Gurmeet and Amrita, 2016). *Pandanus fascicularis* fruits have been reported for their analgesic, antipyretic, anti-inflammatory, antimicrobial and antidiarrheal activity (Baul et al., 2017; Prakash et al., 2016). Many studies have reported the presence of bioactive compounds such as flavonoids, alkaloids, and phenolics, known for their antibacterial effects. Additionally, extracts from this plant have demonstrated antimicrobial activity against various bacterial strains, though their synergy with antibiotics remains unexplored (Gurmeet and Amrita, 2016). Although research has been conducted regarding the antimicrobial properties of this particular part of the plant, no studies have been performed to investigate its potential synergistic effects in conjunction with existing antibiotics to address antibiotic resistance.

**Figure 1:**
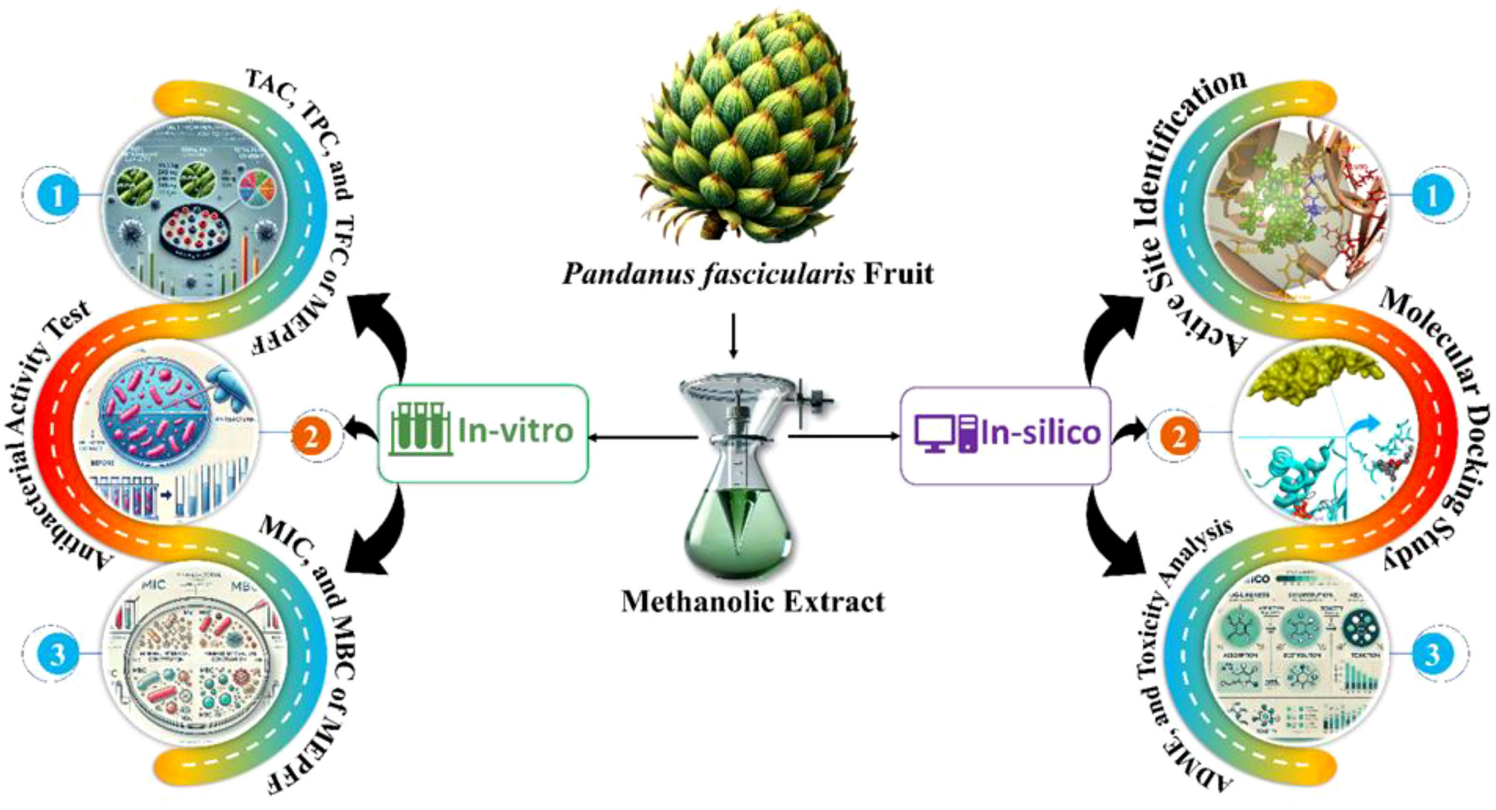
A graphical representation of the workflow outlining the study’s methodology.

We subsequently assessed the efficacy of previously reported compounds using GC-MS against Macrolide 2’-phosphotransferase type II proteins by conducting molecular docking studies and analyzing their in-silico drug-likeness properties. Molecular docking studies are invaluable in the development of new antibacterial drugs, especially against resistant pathogens. This computational technique enables the prediction of binding affinities between drug candidates and bacterial targets, significantly accelerating drug discovery while reducing costs associated with experimental assays (Baothman and Islam, 2024; Kitchen et al., 2004). Docking studies can identify promising compounds by modeling how molecules interact with specific bacterial proteins, enabling targeted inhibition of essential bacterial processes (Morris and Lim-Wilby, 2008). Such targeted approaches are essential in the fight against antibiotic resistance, as they allow for the design of drugs (Barden et al., 2024) with increased efficacy and reduced off-target effects (Ferreira et al., 2015). Additionally, docking facilitates structure-based drug design, allowing researchers to refine molecules for optimal binding and activity before *in vitro* testing (Meng et al., 2011). Consequently, molecular docking is a critical tool in discovering novel anti-bacterial agents that can address the pressing global issue of antibiotic resistance. By exploring these synergistic interactions, our study aims to contribute to the development of more effective treatments for resistant infections. **Figure 1** illustrates a brief methodological overview of the work.

## 2 Results

### 2.1 Phytochemical screening

The methanolic extract of *Pandanus fascicularis* fruits (MEPFF) demonstrated the presence of various bioactive compounds (**Table 1**), including terpenoids, flavonoids, tannins, phenols, cardiac glycosides, saponins, and alkaloids, which collectively suggest a potential for antibacterial activity against resistant clinical strains. Terpenoids and phenolic compounds, known for their antimicrobial and antioxidant properties, can disrupt microbial cell membranes, enhancing the extract’s efficacy against resistant bacteria. Flavonoids and tannins have been widely recognized for their roles in inhibiting bacterial growth by interacting with essential microbial enzymes and cellular structures (Cushnie and Lamb, 2005). Alkaloids enhance MEPFF’s potential by inhibiting bacterial growth through DNA intercalation and enzyme inhibition (Harvey et al., 2015). Saponins also increase cell permeability and exhibit antibacterial properties, potentially amplifying the effects of other compounds (Morrissey and Osbourn, 1999). The absence of sterols in MEPFF suggests a phytochemical profile focused on antibacterial compounds. This composition and in-silico analyses indicate that MEPFF may be an effective source of natural compounds against resistant clinical strains. These findings align with current research trends, aiming to identify plant-derived antimicrobials as an alternative to conventional antibiotics in the fight against antibiotic resistance (Sibanda and Okoh, ).

**Table 1:** List of Phytochemicals present in MEPFF (‘+’ denotes presence and ‘−’ denotes absence).

Sl. No.	Phytochemical components	Results
1.	Terpenoids	**+**
2.	Flavonoids	**+**
3.	Tannins	**+**
4.	Phenols	**+**
5.	Cardiac Glycosides	**+**
6.	Saponins	**+**
7.	Alkaloids	**+**
8.	Sterols	**−**

### 2.2 Antioxidant activity test

**Figure 2:**
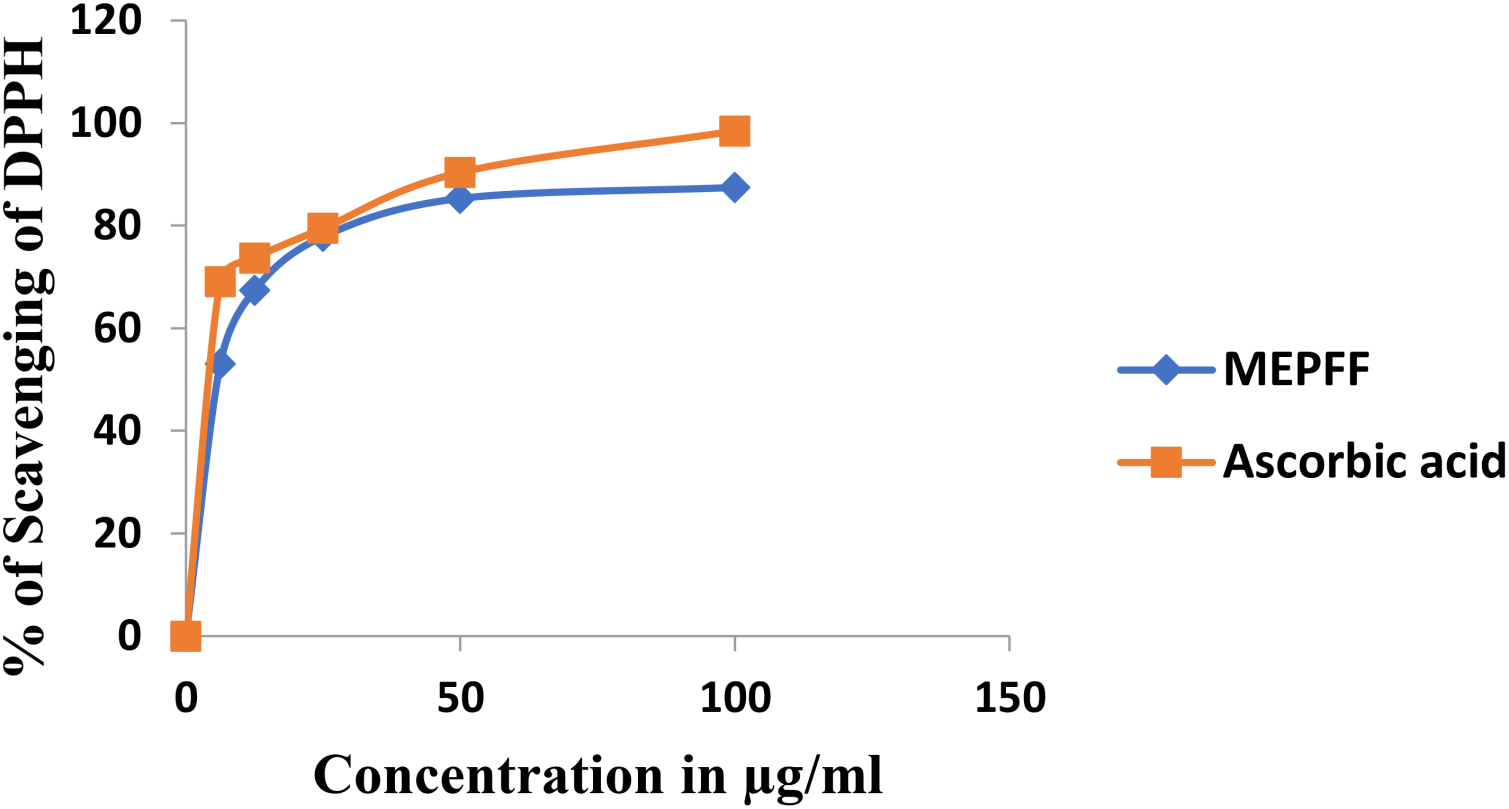
Percentage of DPPH scavenging of MEPFF and the standard ascorbic acid.

The antioxidant efficacy of the methanolic extract of *Pandanus fascicularis* fruits (MEPFF) was assessed using the DPPH scavenging assay, with results illustrated in **Figure 2**. The extract and ascorbic acid exhibited concentration-dependent rises in scavenging capacity of DPPH. The IC_50_ of the MEPFF and standard ascorbic acid in DPPH assay were recorded 12.13 ± 0.63 µg/ml and 2.81 ± 0.91 µg/ml respectively.

Both MEPFF and the standard ascorbic acid demonstrated a concentration-dependent increase in DPPH scavenging activity. As shown, the percentage of DPPH scavenging for both MEPFF and ascorbic acid rose sharply at lower concentrations, eventually plateauing as concentrations approached higher levels, indicating effective free radical scavenging properties for both. These findings align with previous reports highlighting the antioxidant properties of plant extracts due to their phytochemical constituents (Brand-Williams et al., 1995). The IC_50_ values further support this antioxidant potential, where MEPFF exhibited an IC_50_ of 12.13 ± 0.63 µg/ml, compared to a significantly lower IC_50_ for ascorbic acid at 2.81 ± 0.91 µg/ml, as presented in **Table 2**. The lower IC_50_ for ascorbic acid suggests it is more potent as a radical scavenger than MEPFF, possibly due to its pure and concentrated nature, while MEPFF’s activity is influenced by the complexity and concentration of its bioactive compounds (Velioglu et al., 1998). These results corroborate the use of MEPFF as a natural antioxidant agent, though its efficacy is less than that of the standard ascorbic acid, which is consistent with known antioxidant benchmarks in phytochemical studies (Wong et al., 2006).

**Table 2:** IC_50_ value of MEPFF and the standard ascorbic acid.

Sample	IC_50_ (µg/ml)
MEPFF	12.13 ± 0.63
Ascorbic acid	2.81 ± 0.91

### 2.3 Total antioxidant capacity, total phenol content and total flavonoid content of MEPFF

The total antioxidant capacity (TAC), total phenol content (TPC), and total flavonoid content (TFC) of MEPFF were determined using standard methods. The results showed that MEPFF contained 183 ± 9.54 mg of quercetin equivalents (QE) for TFC, 248.33 ± 11.06 mg of gallic acid equivalents (GAE) for TPC, and 95.33 ± 8.33 mg of ascorbic acid equivalents (AAE) per gram of dry extract (**Table 3**). These findings suggest that MEPFF is rich in bioactive compounds, which may contribute to its therapeutic efficacy, particularly against multidrug-resistant (MDR) pathogens. The antioxidant and polyphenolic content observed in MEPFF aligns with the established role of these compounds in enhancing antibacterial activity and reducing oxidative stress (Irshad et al., 2025). Notably, similar studies on other plant extracts, such as those of Cinnamomum verum (Ul Hasnain et al., 2024) and Aloe vera (Yoruk and Istanbullu Paksoy, 2024), have also demonstrated a strong correlation between higher phenolic and flavonoid concentrations and increased antimicrobial and antioxidant activities. These bioactive compounds not only play a critical role in inhibiting microbial growth but also mitigate the effects of oxidative stress, reinforcing the therapeutic potential of MEPFF (Tungmunnithum et al., 2018).

**Table 3:** Total antioxidant capacity, total phenol content and total flavonoid content of MEPFF.

Total antioxidant capacity (mg of AAE/gm of extract)	Total phenol content (mg of GAE/gm of extract)	Total flavonoid content (mg of QE/gm of extract)
95.33 ± 8.33	248.33 ± 11.06	183 ± 9.54

### 2.4 Antibacterial activity test by disc diffusion method

Based on the disc diffusion method results, the methanolic extract from *Pandanus fascicularis* fruits (MEPFF) showed notable antibacterial activity against various resistant bacterial strains. For instance, MEPFF alone at 400 µg/disc demonstrated significant inhibition zones against *Staphylococcus aureus* (12.67 ± 1.15 mm, p < 0.05), *Bacillus cereus* (13.33 ± 0.58 mm, *p < 0.01), and *Escherichia coli* (15 ± 1.00 mm, p < 0.05) when compared to azithromycin (10 µg/disc), which displayed lower inhibition zones across all tested strains (Dawood et al., 2022) (**Table 4**). Notably, the combination of MEPFF (200 µg/disc) with azithromycin (5 µg/disc) produced significantly larger inhibition zones, particularly against *Staphylococcus aureus* (24.17 ± 2.36 mm, **p < 0.001), *Bacillus cereus* (23.33 ± 2.08 mm, **p < 0.001), *Escherichia coli* (21.77 ± 1.37 mm, **p < 0.001), and *Pseudomonas aeruginosa* (22.33 ± 2.08 mm, **p < 0.001), indicating enhanced synergistic effects (Angelini, 2024). These findings align with prior research suggesting the efficacy of plant extract-antibiotic combinations against resistant strains (Anand and Rai, 2017). The results indicate a potential for combined therapy involving MEPFF and azithromycin to combat resistant bacterial infections effectively (Hossain et al., 2021).

**Table 4:** Antibacterial activity of MEPFF and azithromycin combined with MEPFF.

Sample	Diameter of zone of inhibition (mm)			
	*Staphylococcus aureus*	*Bacillus cereus*	*Escherichia coli*	*Pseudomonas aeruginosa*
MEPFF (400 µg/disc)	12.67 ± 1.15^*^	13.33 ± 0.58^**^	15 ± 1.00^*^	11.33 ± 1.53^ns^
Azithromycin (10 µg/disc)	8.83 ± 1.25	8.67 ± 1.53	11.53 ± 0.5	10.17 ± 0.76
MEPFF (200 µg/disc) and Azithromycin (5 µg/disc)	24.17 ± 2.36^***^	23.33 ± 2.08^***^	21.77 ± 1.37^***^	22.33 ± 2.08^***^

Assay was performed in triplicate and the results are the mean of three values ± SD. Data was analyzed by two-way ANOVA followed by Bonferroni post test. ***p < 0.001, **p < 0.01, * p < 0.05; compared to azithromycin group.

### 2.5 Minimum inhibitory concentration and minimum bactericidal concentration of MEPFF alone and in combination with azithromycin

**Figure 3:**
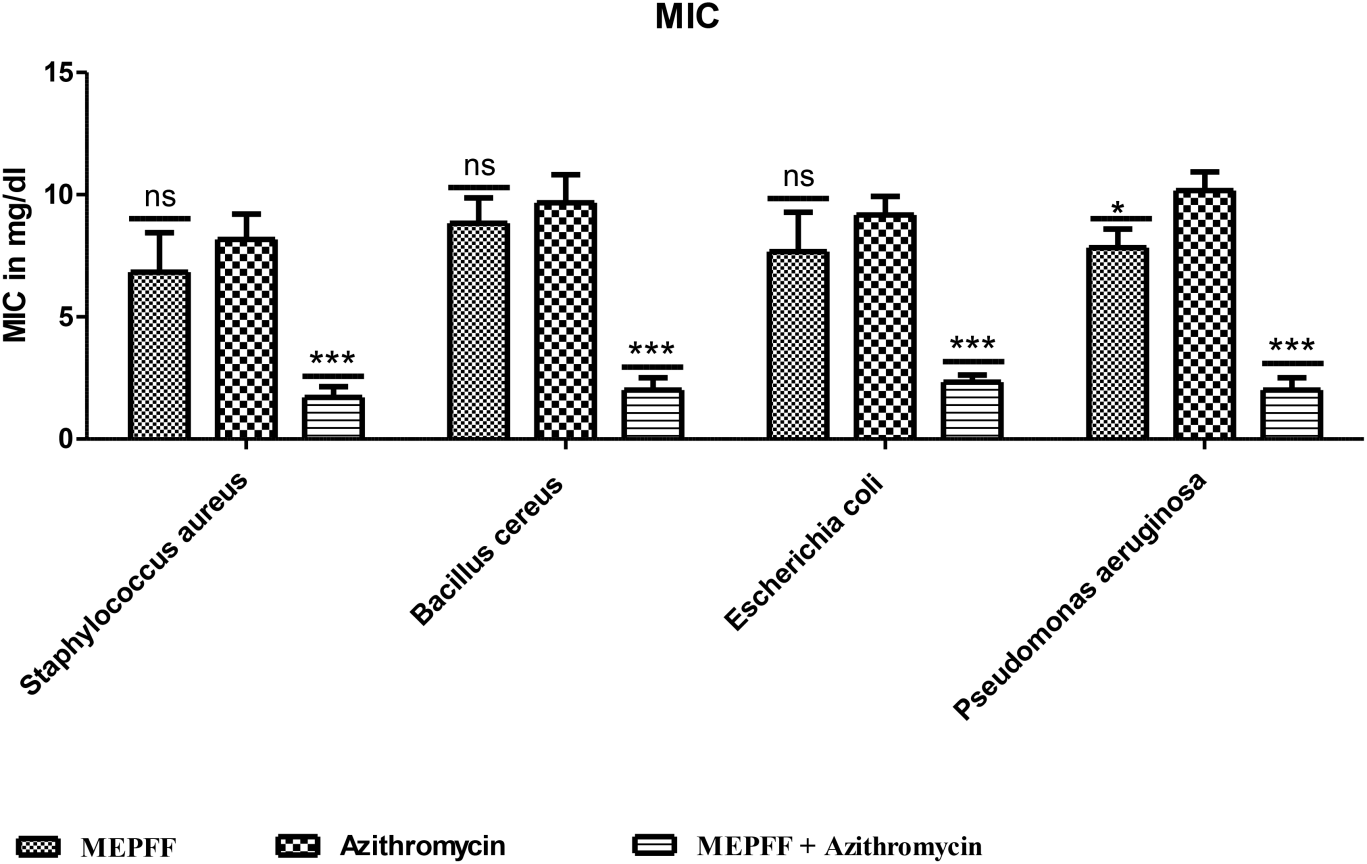
MIC of MEPFF alone and in combination with azithromycin (where 'ns' indicates non significant, '*' indicates p < 0.05, and '***' indicates p < 0.001).

**Figure 4:**
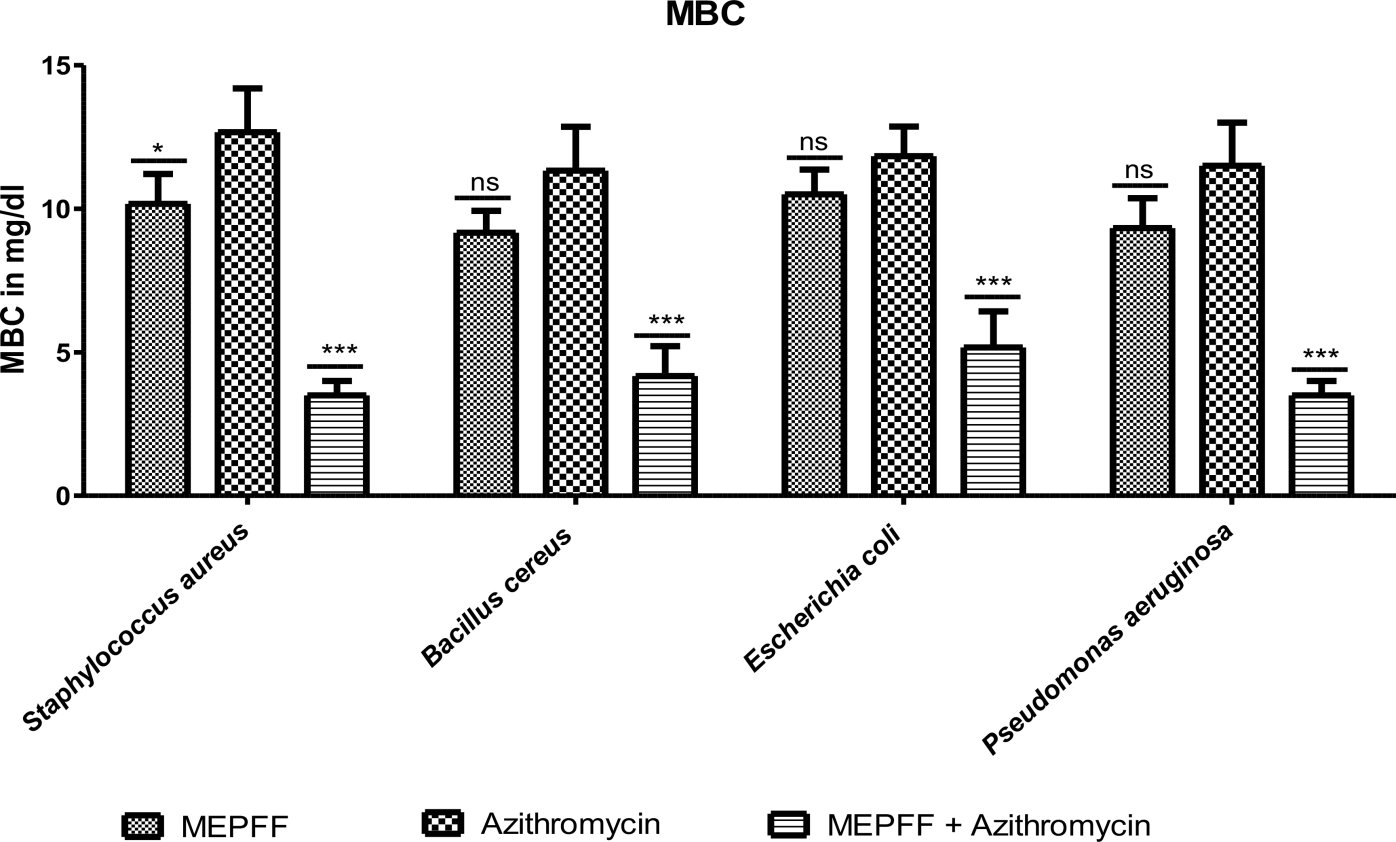
MBC of MEPFF alone and in combination with azithromycin (where 'ns' indicates non significant, '*' indicates p < 0.05, and '***' indicates p < 0.001).

The MEPFF showed no significant MIC and MBC values against all tested bacteria compared to azithromycin itself but both MIC and MBC results were demonstrated significant (***p < 0.001) compared to azithromycin when tested by the combination of azithromycin with MEPFF (**Figures 3**, **4**). MEPFF alone exhibited no significant difference in MIC values against *Staphylococcus aureus*, *Bacillus cereus*, and *Escherichia coli* compared to azithromycin (ns, p > 0.05), indicating limited efficacy when used independently. However, combining MEPFF with azithromycin significantly reduced the MIC values against *Bacillus cereus, Escherichia co*li, and *Pseudomonas aeruginosa* (**p < 0.001), compared to azithromycin alone, demonstrating enhanced antibacterial potency. Specifically, this combination led to substantial reductions in MIC values, supporting a potential synergistic interaction that enhances azithromycin’s efficacy against resistant strains (Angelini, 2024). These findings underscore the potential of MEPFF to bolster conventional antibiotics and improve treatment outcomes against resistant infections. The assay was performed in triplicate, and the results are the mean of three values ± SD. Data was analyzed by two-way ANOVA followed by Bonferroni post test. ***p < 0.001, *p < 0.05; compared to azithromycin group. ns represents non-significant.

**Figure 4** demonstrated the results for the Minimum Bactericidal Concentration (MBC) of MEPFF alone and in combination with azithromycin against various pathogens. The data indicates that MEPFF, when combined with azithromycin, significantly reduced the MBC values for *Staphylococcus aureus*, *Bacillus cereus*, *Escherichia coli*, and *Pseudomonas aeruginosa* (p < 0.001), highlighting its enhanced bactericidal effects. Specifically, the combination treatment demonstrated superior bactericidal activity compared to azithromycin alone, where the MBC remained relatively higher for most strains (ns, p > 0.05) when MEPFF was used alone. This suggests a synergistic effect between MEPFF and azithromycin in combating resistant bacterial strains. The assay was performed in triplicate, with results representing the mean ± standard deviation. Statistical analysis was conducted using two-way ANOVA followed by Bonferroni’s post-test, showing a significant reduction in MBC values for the combination group (***p < 0.001, *p < 0.05). These findings align with similar studies where natural products enhance antibiotic efficacy, supporting their potential in combating multidrug-resistant pathogens (Hashim et al., 2025; Shang et al., 2024).

### 2.6 MBC/MIC ratio

**Figure 5:**
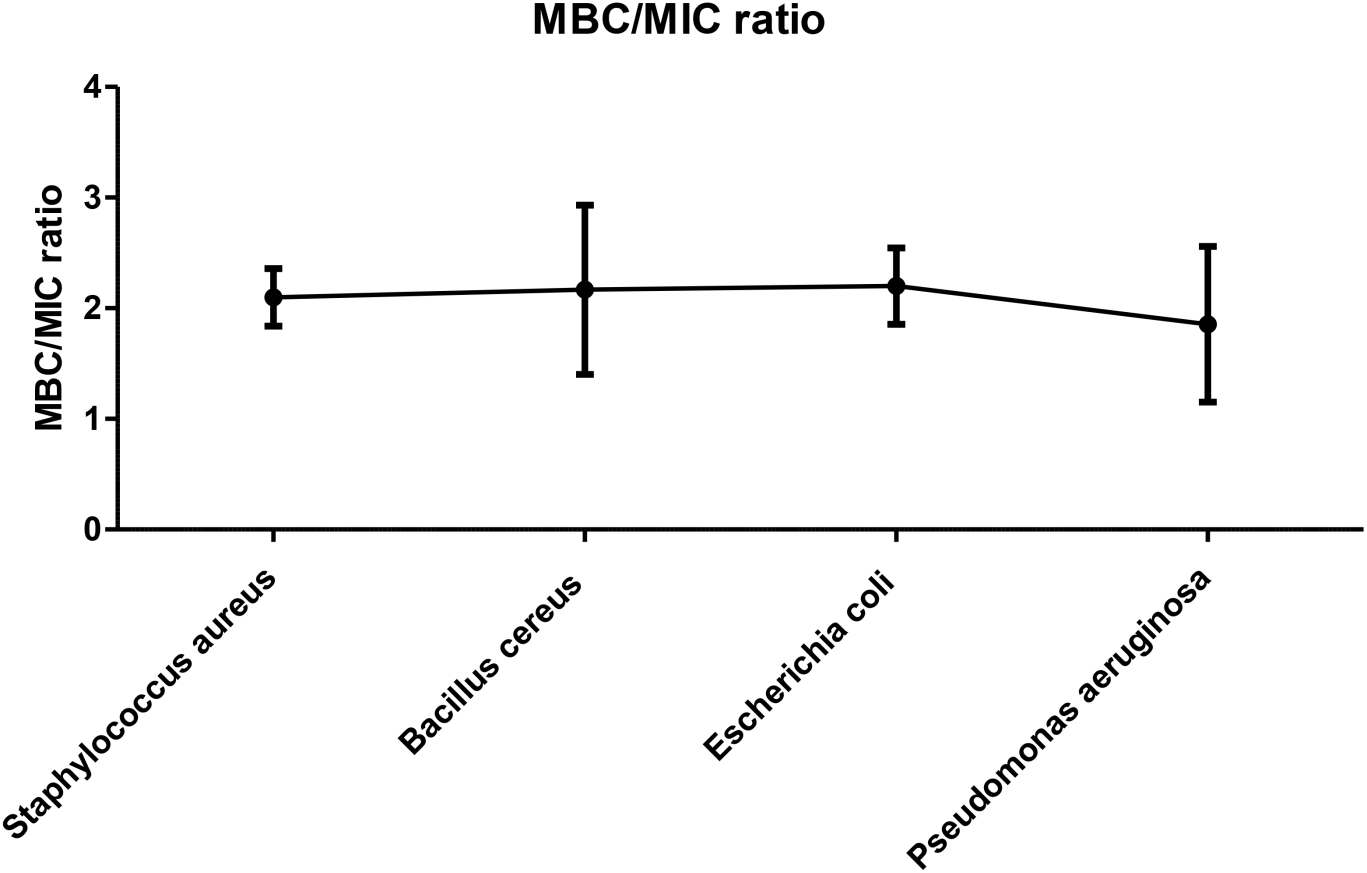
MBC/MIC ratio of MEPFF in combination with azithromycin. The results are the mean of three values ± SD.

Based on our study results shown in the **Figure 5**, the MBC/MIC ratios of MEPFF in combination with azithromycin for various bacterial strains, including *Staphylococcus aureus*, *Bacillus cereus*, *Escherichia coli*, and *Pseudomonas aeruginosa*, all remain below 4, indicating bactericidal activity. According to the classification system by Levison (2004), a MBC/MIC ratio less than 4 suggests that the treatment is bactericidal, effectively killing the bacteria rather than merely inhibiting their growth (Angelini, 2024).

The figure confirms this pattern, as all the tested strains exhibited MBC/MIC ratios consistently under the threshold, thereby suggesting that MEPFF, in combination with azithromycin, has potent bactericidal effects against these MDR pathogens. These findings are consistent with similar studies on plant extracts combined with antibiotics, where such combinations often enhance the bactericidal effects and reduce the possibility of resistance development (Vui et al., 2025). By lowering the MBC/MIC ratio, the combination treatment demonstrates its potential as an effective strategy in combating multidrug-resistant bacterial infections, further validating the growing interest in plant-antibiotic synergy (Angelini, 2024).

### 2.7 Fractional inhibitory concentration index

**Figure 6:**
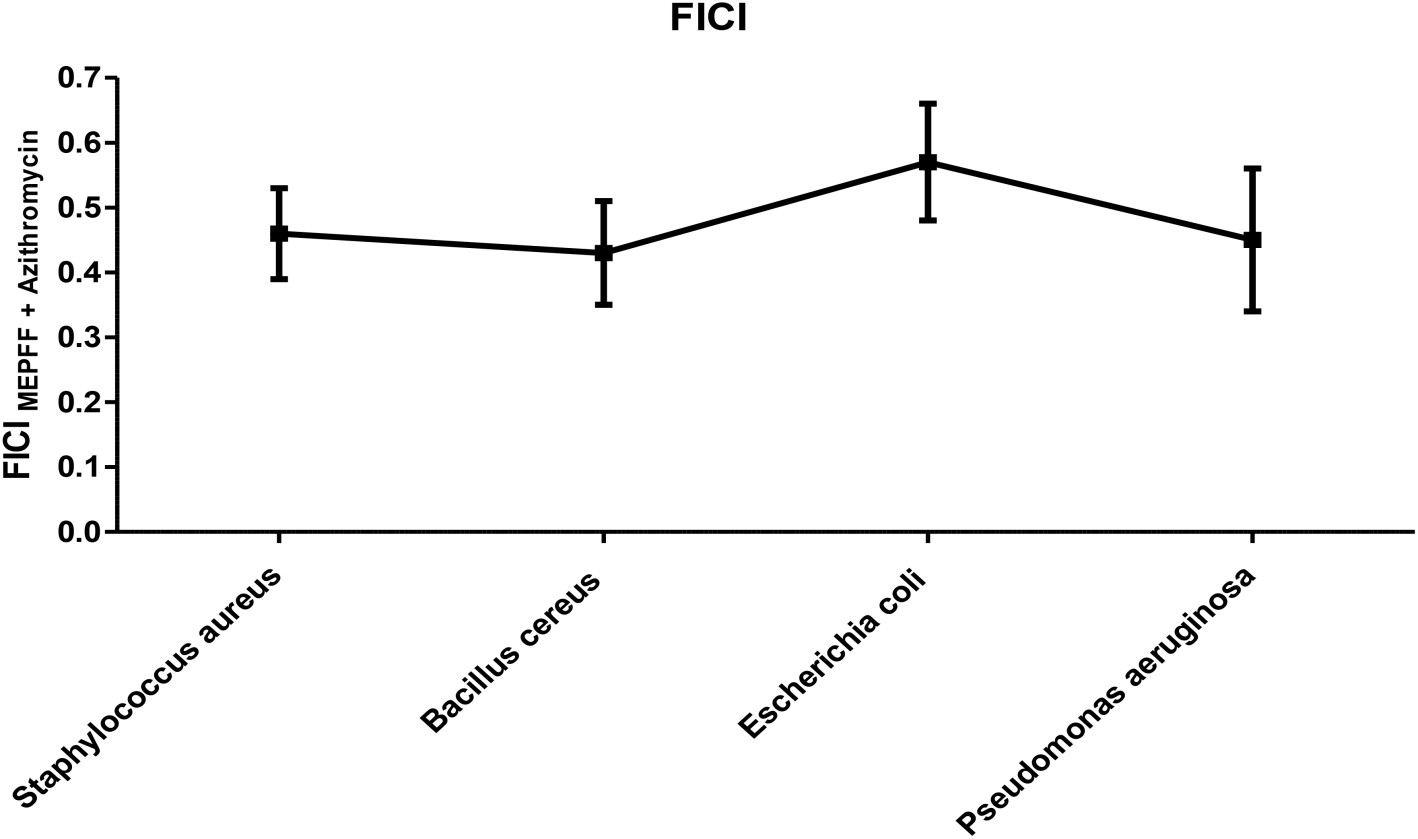
FICI of MEPFF in combination with azithromycin. The results are the mean of three values ± SD.

MEPFF in combination with azithromycin exhibited synergism activity against *Staphylococcus aureus, Bacillus cereus* and *Pseudomonas aeruginosa* but against *E. coli* provided partial synergistic activity (**Figure 6**). The fractional inhibitory concentration index (FICI) for MEPFF combined with azithromycin demonstrated synergistic antibacterial effects against *Staphylococcus aureus*, *Bacillus cereus*, and *Pseudomonas aeruginosa*, as indicated by FICI values below 0.5. Against *Escherichia coli*, the combination exhibited partial synergism, with FICI values between 0.5 and 1, suggesting a less pronounced interaction. These results suggest that MEPFF, when combined with azithromycin, can enhance the antibacterial efficacy of azithromycin particularly against certain resistant strains. The FICI analysis reinforces the potential utility of MEPFF as an adjunct in antibiotic therapies to combat resistant bacteria.

### 2.8 Active sites identification and grid box generation

**Figure 7:**
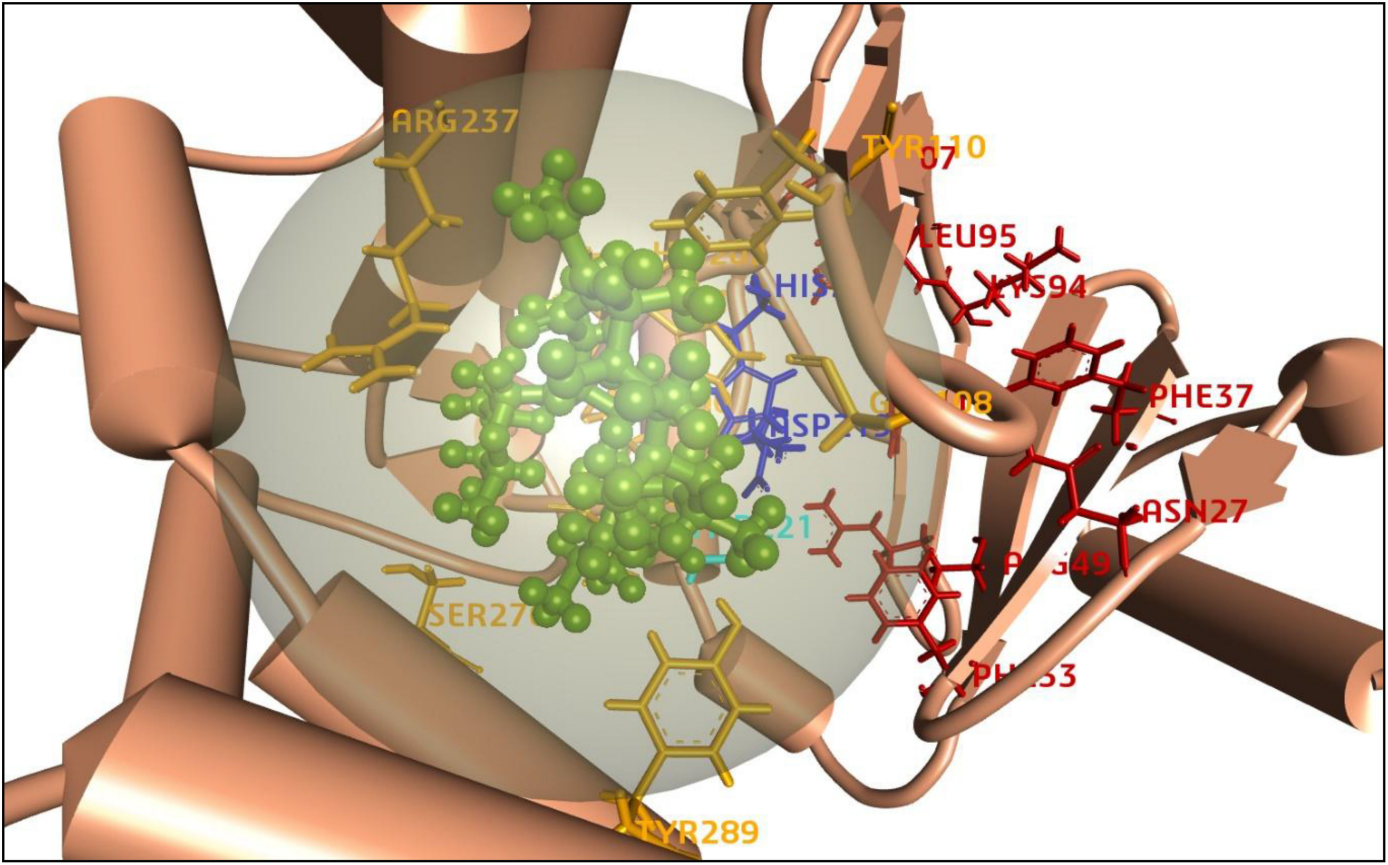
Representation of the 3D structure of the active sites of the target protein. The protein backbones are colored in darker orange, active sites are labeled as AS1 (HIS205, ASP219) in blue, AS2 (THR221) in light blue, AS3 (LEU95, LYS94, PHE37, ASN27, ARG49, PHE33, MET207) in red, and AS4 (ARG237, TYR110, GLY108, TYR289, SER276, HIS202, VAL201, ASP200, GLU222) in yellow. The azithromycin ligand is depicted in green as a ball-and-stick model.

A protein’s active site is a particular area where interactions with ligands take place, and it is essential for molecular docking. It usually contains amino acid residues with shape, electrostatics, and complementarity that promote binding. During analysis of the protein-ligand complex (PDB: 5IGV), this study identified a total of four binding sites (AS1, AS2, AS3, and AS4) to proceed with the molecular docking process and shown in **Figure 7**. In addition, to initiate the molecular docking procedure using PyRx, a grid-based docking tool, it is essential to define the size of the receptor grid box. Before commencing the molecular docking procedure, fixing the grid box helps improve the accuracy of scoring for the ligand poses. Therefore, our study has generated a receptor grid box with dimensions X=−10.863, Y=−10.825, and Z=26.200, which were previously studied for site locations.

### 2.9 Molecular docking study

By anticipating the compound’s binding interactions with target proteins, molecular docking accurately assists in identifying possible drug candidates. This makes it possible for researchers to quickly screen through vast libraries of chemicals and identify those that have the best chance of being therapeutically effective (Morris et al., 2009). Therefore, molecular docking was conducted on 16 compounds from *Pandanus fascicularis*to to assess their binding affinity for the Mph-II protein, listed in **Supplementary Table S1**. Among them, pandamarilactone-1 (CID-102224960), norpandamarilactone-B (CID-11073796), and thiamine (CID-1130) exhibited the most favorable binding poses with energies of −9.9 kcal/mol, −8.9 kcal/mol, and −8.5 kcal/mol, respectively, shown in **Table 5**.

**Table 5:** List of the top three compounds and their chemical name, molecular formula, binding affinity (kcal/mol).

Chemical name & molecular formula	Structure	Binding affinity (kcal/mol)
Pandamarilactone-1 C_18_H_23_NO_4_	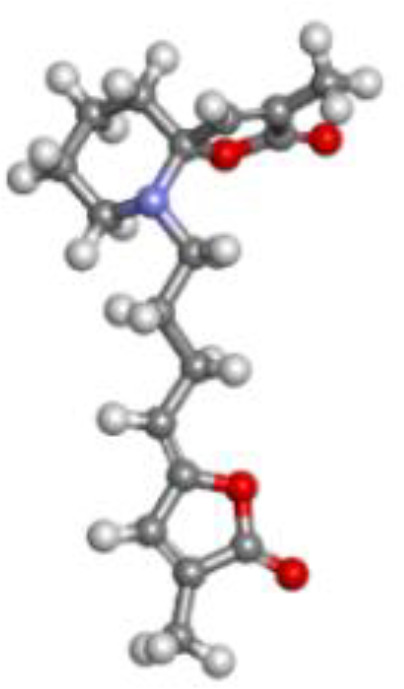	−9.9
Norpandamarilactone-B C_9_H_13_NO_2_	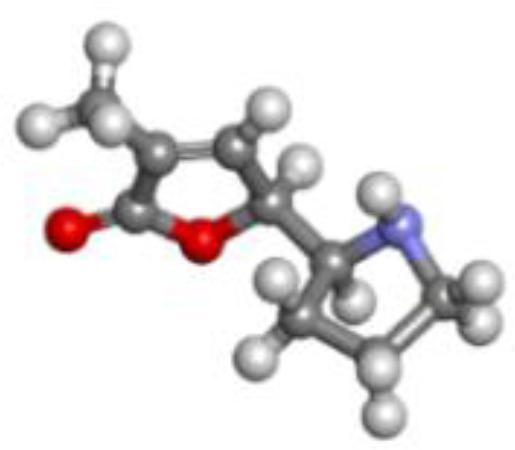	−8.9
Thaimine C_12_H_17_N_4_OS^+^	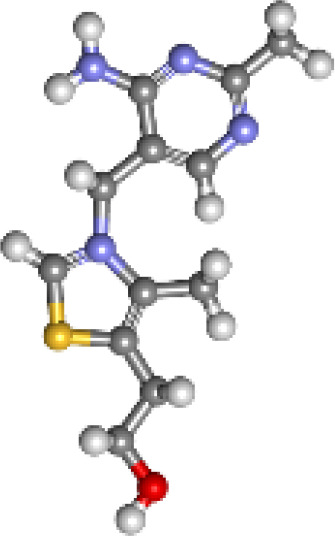	−8.5

**Figure 8:**
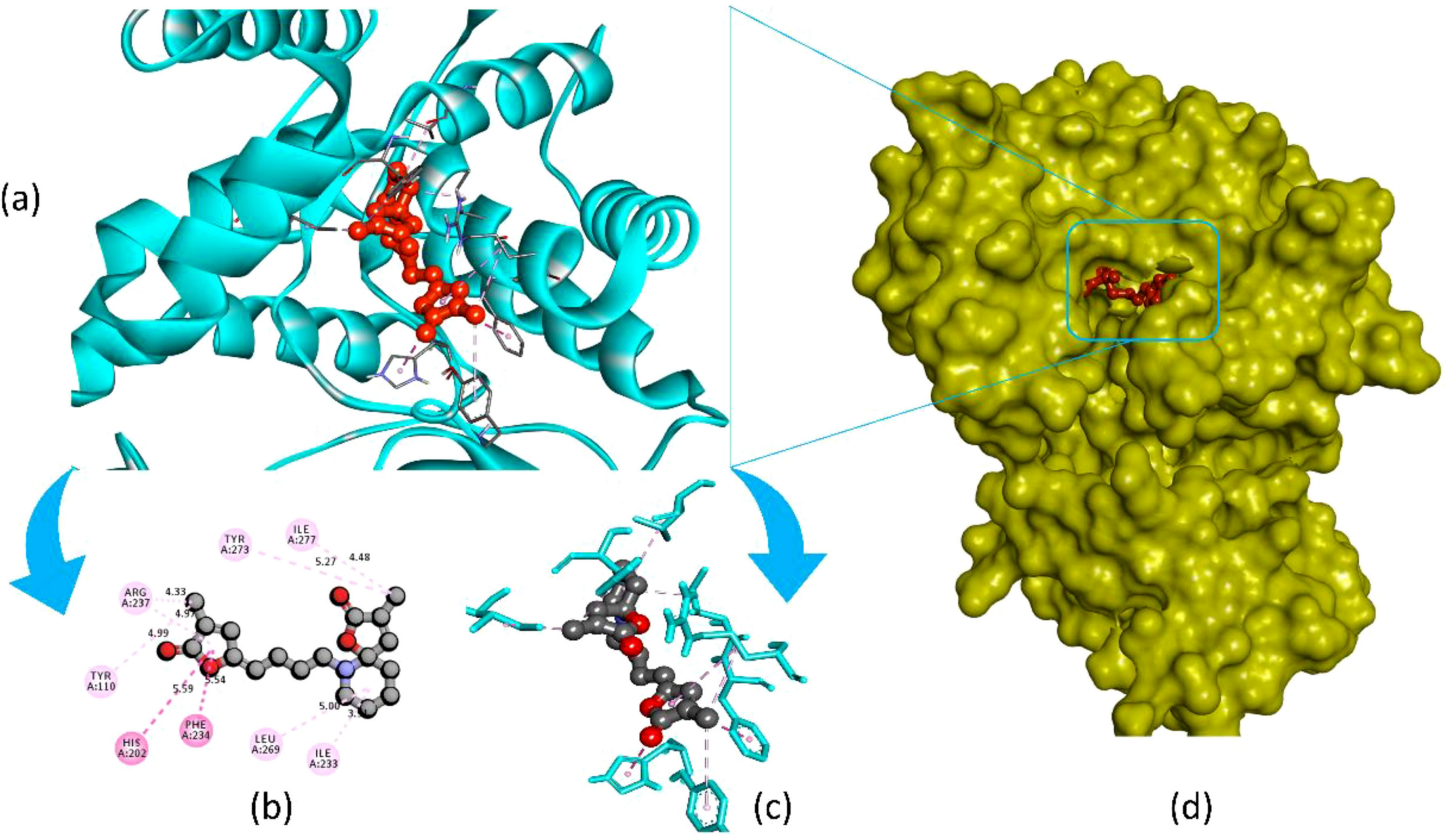
Illustrates the molecular docking of the MPh-II (Macrolide Phosphotransferase-II) protein (PDB ID: 5IGV) from *E. coli* with compound pandamarilactone-1. Panel **(a)** depicts the cartoon view, while panels **(b, c)** show the 2D view and panel **(d)** presents the surface view of the protein-ligand complex.

**Figure 9:**
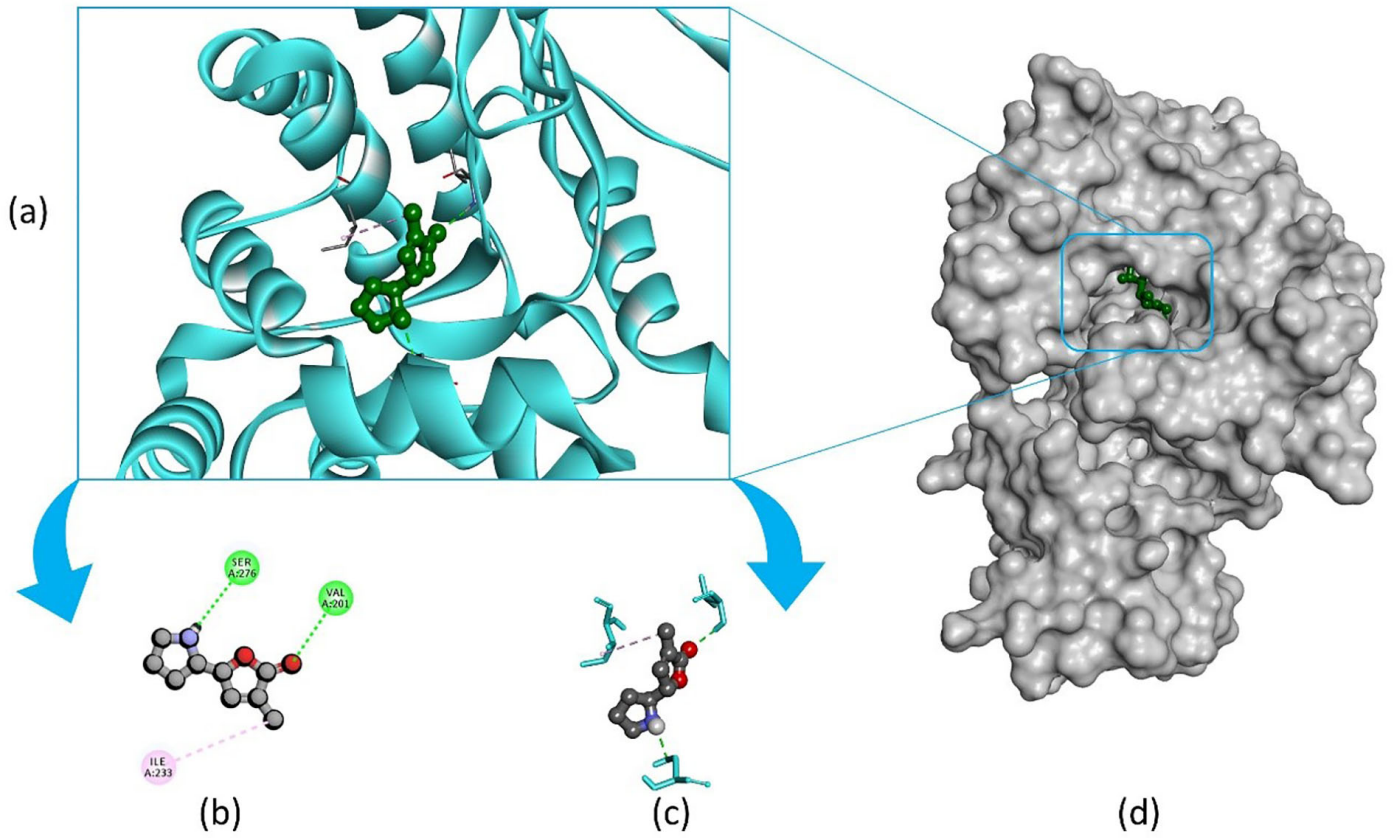
Illustrates the molecular docking of the MPh-II (Macrolide Phosphotransferase-II) protein (PDB ID: 5IGV) from *E. coli* with compound norpandamarilactone-B. Panel **(a)** depicts the cartoon view, while panels **(b, c)** show the 2D view and panel **(d)** presents the surface view of the protein-ligand complex.

**Figure 10:**
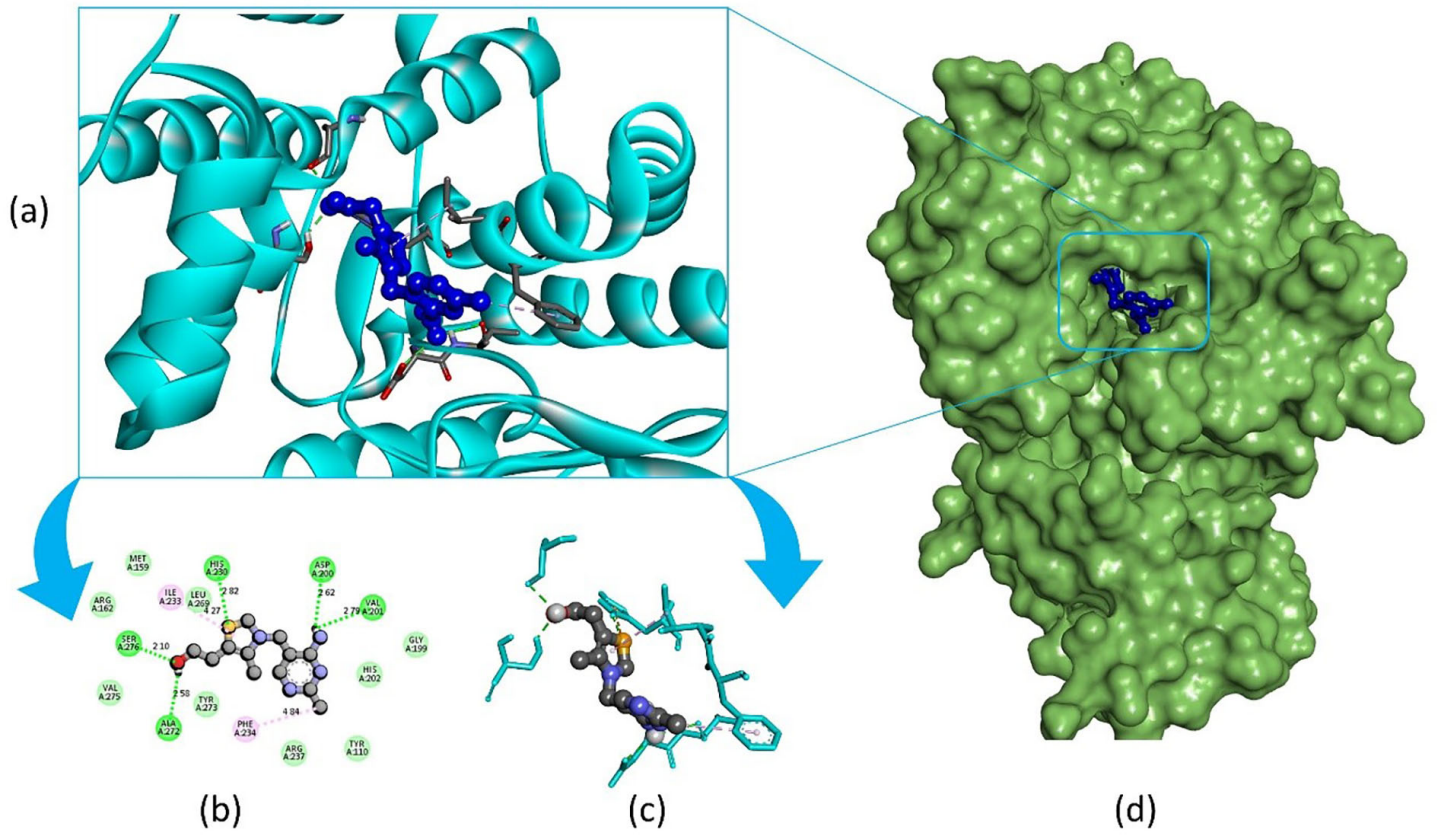
Illustrates the molecular docking of the MPh-II (Macrolide Phosphotransferase-II) protein (PDB ID: 5IGV) from *E. coli* with the compound thiamine. Panel **(a)** depicts the cartoon view, while panels **(b)** and **(c)** show the 2D view and panel **(d)** presents the surface view of the protein-ligand complex.

Pandamarilactone-1 compound was stabilized with Mph-II protein by making three hydrophobic bonds and one alkyl bond at ile277, try273, leu269, and ile233; it also had pi-alkyl and pi-pi stacked interactions with arg237, tyr110, his202, and phe234; did not exhibit any hydrogen bond interactions (**Figure 8**; **Table 6**). The compound norpandamarilactone-B and the target protein formed two conventional hydrogen bonds with ser276 and val201 and one alkyl bond with ile233 residues; they did not exhibit any pi-alkyl interactions (**Figure 9**, **Table 6**). Five hydrogen bonds stabilized the compound thiamine and MPh-II protein complex at his230, ser276, ala272, asp200, and val201, one pi-sulphur bond at his236, one pi-alkyl bond phe234, and one alkyl bond at ile233 (**Figure 10**). According to *in silico* analysis, norpandamarilactone-B and thiamine, with more hydrogen bonds crucial for stabilizing protein-ligand complexes (Dill, 1990), emerge as the top phytocompounds and potential antibacterial agents.

**Table 6:** List of amino acid integrations of three (pandamarilactone-1, norpandamarilactone-B, and thiamine) phytocompounds with MPh-II protein.

Compound	Interaction of amino acid residues	Distance (Å)	Bond category	Bond types
Pandamarilactone-1	HIS202	5.58659	Hydrophobic	Pi-Pi Stacked
	PHE234	5.53615	Hydrophobic	Pi-Pi T-shaped
	ILE233	3.90811	Hydrophobic	Alkyl
	LEU269	5.00137	Hydrophobic	Alkyl
	ILE277	4.47566	Hydrophobic	Alkyl
	ARG237	4.33457	Hydrophobic	Alkyl
	TYR110	4.99202	Hydrophobic	Pi-Alkyl
	TYR273	5.27234	Hydrophobic	Pi-Alkyl
	ARG237	4.96541	Hydrophobic	Pi-Alkyl
Norpandamarilactone-B	VAL201	2.69811	Hydrogen Bond	Conventional Hydrogen Bond
	SER276	2.35359	Hydrogen Bond	Conventional Hydrogen Bond
	ILE233	4.93567	Hydrophobic	Alkyl
Thiamine	HIS230	2.81874	Hydrogen Bond	Conventional Hydrogen Bond
	SER276	2.1006	Hydrogen Bond	Conventional Hydrogen Bond
	ALA272	2.57706	Hydrogen Bond	Conventional Hydrogen Bond
	ASP200	2.61676	Hydrogen Bond	Conventional Hydrogen Bond
	VAL201	2.79424	Hydrogen Bond	Conventional Hydrogen Bond
	HIS230	4.67195	Other	Pi-Sulfur
	ILE233	4.2704	Hydrophobic	Alkyl
	PHE234	4.8385	Hydrophobic	Pi-Alkyl

### 2.10 *In silico* drug-likeness, ADME, and toxicity prediction

To reduce the chances of failure in clinical and preclinical trials, predicting pharmacokinetic behavior, optimizing lead compounds, and lowering experimental costs all depend on analyzing drug-likeness and ADME (Absorption, Distribution, Metabolism, Excretion) features in in-silico drug discovery research (Asseri et al., 2024). All three phytocompounds adhere to Lipinski’s Rule of Five and do not exhibit Veber or Ghose violations, whereas phytocompound pandamarilactone-1 has lead-like rule violations. High GI and intestinal absorption indicate efficient drug uptake and bioavailability, enhancing efficacy (Chow, 2014). Our compounds exhibited very high GI and intestinal absorption. In toxicity prediction, norpandamariclactone-B and thiamine showed inactivity against Ames, carcinogenicity (mouse and rat), TA100_10RLI, fish toxicity, and skin sensitization, as shown in **Table 7**. Additionally, pandamarilactone-1 and thiamine were effective against honeybee toxicity except for compound norpandamarilactone-B. Based on toxicity-predicted data, pandamarilactone-1 and thiamine appear to be the best compounds due to their effectiveness against honeybee toxicity.

**Table 7:** Drug-likeness, ADME, and toxicity prediction details of three (pandamarilactone-1, norpandamarilactone-B, and thiamine) phytocompounds.

Drug likeness prediction	Pandamarilactone-1	Norpandamarilactone-B	Thiamine
Rule of five	Suitable	Suitable	Suitable
Lead like rule violations	1	0	1
Rule of five violations	0	0	0
CMC like rule	Qualified	Qualified	Failed
CMC like rule violations	0	0	1
MDDR like rule	Mid-structure	Mid-structure	Mid-structure
MDDR like rule violation fields	No rotatable bonds	No rings	No rings
MDDR like rule violations	1	2	2
WDI like rule	In 90% cutoff	In 90% cutoff	Failed
WDI like rule violations	0	0	0
WDI like rule violation fields	0	0	Failed
Lipinski violations	0	0	0
Veber violations	0	0	0
Ghose violations	0	0	0
ADME prediction (absorption and distribution)			
GI absorption	High	High	High
Intestinal absorption	93.47%	92.65%	100%
LogP (o/w)	2.92	1.08	0.53
Surface area (Å^2^)	55.84	38.33	104.15
HIA	98.37%	93.25	91.11%
Caco-2 (nm/sec)	46.07	23.94	5.44
MDCK	0.47	21.45	0.55
Skin permeability	−3.44	−3.05	−4.57
BBB penetration	0.06	0.48	0.03
Plasma protein binding (%)	84.49	55.21	9.07
CYP3A4 inhibition	No	No	No
Excretion prediction			
Total clearance (ml/min/kg)	1.115	1.078	1.056
Renal OCT2 substrate	No	No	No
Toxicity prediction			
Ames_test	Nontoxic	Nontoxic	Nontoxic
TA100_10RLI	Negative	Negative	Negative
TA100_NA	Negative	Positive	Negative
TA1535_10RLI	Negative	Positive	Positive
TA1535_NA	Negative	Positive	Positive
Carcinogenicity (Mouse)	Positive	Negative	Negative
Carcinogenicity (Rat)	Positive	Negative	Negative
Fish toxicity	Low	Low	Low
Honeybee toxicity	Low	High	Low
Hepatotoxicity	No	Yes	Yes
Skin sensitization	No	No	No

## 3 Discussion

Bioactive molecules effectively treat diseases caused by pathogenic bacteria. They are primarily sourced from medicinal plants (Ralte et al., 2022; Vanitha et al., 2020), as more than 80% of people rely on herbal medicine for basic healthcare needs (Kebede et al., 2021). The biological activities of natural chemicals from various sources, including plants, have been characterized. Their antibacterial activity is particularly noteworthy because of the rise in antibiotic resistance and the emergence of novel pathogen strains. Utilizing plant extracts or isolated chemicals as essential tools to restore the susceptibility of resistant microbes and to boost antibiotic action is a promising strategy (Acharjee et al., 2023; Chauhan et al., 2023). The successful repurposing of current antibiotics for treating and preventing infections require a combination of several natural compounds and a thorough analysis of their biological activity (Chauhan et al., 2023).

Bioactive medicinal plants possess therapeutic properties such as antimicrobial, antiviral, antidiabetic, antioxidant, anticancer, memory-enhancing, and cholesterol-lowering effects. Many studies reported that the methanolic extract of *P. fascicularis* leaves contains antimicrobial phytoconstituents like alkaloids and flavonoids. The ethanolic and aqueous extracts of *P. fascicularis* prop roots contain anti-inflammatory compounds such as flavonoids, glycosides, and alkaloids (Panda et al., 2008). Additionally, the chloroform extract of leaves contains steroids, terpenoids, flavonoids, saponins, and tannins (Tesaki et al., 1999). In this study, the methanolic extract of *P. fascicularis* fruits contained seven types of phytoconstituents: terpenoids, flavonoids, tannins, phenols, glycosides, saponins, and alkaloids. The synergistic effects of MEPFF were evaluated by measuring the diameter of the inhibition zone in combination with azithromycin antibiotics using disc diffusion methods against four different MDR bacteria. Most importantly, our research found that mixing the extract with azithromycin dramatically improves its ability to kill bacteria compared to using just the extract or the antibiotic against each of the four bacterial strains.

A previous study stated that phenolics were some of the most effective antibacterial compounds (Chang et al., 2001). Phenolic compounds are a class of antioxidant agents due to their redox properties, which allow them to act as reducing agents, hydrogen donors, and singlet oxygen quenchers (Jorgensen and Ferraro, 2000). Flavonoids are a large group of phenolic plant constituents, which show antioxidant activity through scavenging or chelating processes. In this study, MEPFF exhibited the highest DPPH scavenging activity with an IC_50_ value of 12.13 μg/ml. At the same time, standard ascorbic acid showed the lowest activity at 2.81 μg/ml in the DPPH assay. Furthermore, this study determined the TAC, TPC, and TFC of MEPFF, showing inhibition zones against *S. aureus*, *B. cereus*, *E. coli*, and *P. aeruginosa* with diameters of 12.67 ± 1.15 mm, 13.33 ± 1.58 mm, 15 ± 1.00 mm, and 11.33 ± 1.53 mm, respectively, shown in **Table 4**. Azithromycin (10 µg/disc) alone showed zone of inhibition diameters of 8.83 ± 1.25 mm, 8.67 ± 1.53 mm, 11.53 ± 0.5 mm, and 10.17 ± 0.76 mm. Notably, when azithromycin was combined with MEPFF, the zone of inhibition significantly increased against the mentioned bacterial strains. Based on our study data, it is concluded that combining azithromycin with the extract enhances its antibacterial properties against the tested bacteria, as the larger zone of inhibition indicates better activity (Tan and Takayama, 2019). Combining MEPFF with azithromycin significantly enhanced both MIC and MBC outcomes against resistant bacterial strains, demonstrating improved antibacterial efficacy over azithromycin alone. Although MEPFF alone showed no significant reduction in MIC and MBC values against *Staphylococcus aureus*, *Bacillus cereus*, and *Escherichia coli* (ns, *p* > 0.05), its combination with azithromycin markedly decreased these values, reflecting a synergistic effect that could enhance treatment potential for resistant infections (Betoni et al., 2006; de Lima et al., 2006; Nascimento et al., 2000). This synergy is consistent with plant-antibiotic interactions that strengthen bacterial susceptibility (Aiyegoro and Okoh, 2009; Gibbons, 2005). The significantly lower MIC and MBC values (**p < 0.001) with the combination underscore its potential as a practical approach to counteract antibiotic resistance, providing a promising alternative for combating multidrug-resistant strains.

Molecular docking, a software-based technique, evaluates how a protein interacts with potential drugs, aiding in the search for effective molecules. After a literature search, 16 compounds detected through GC-MS analysis of MEPFF were selected for molecular docking studies (Acharjee et al., 2023). We integrated our investigation findings with a method to identify a potential component from *Acanthus ilicifolius* for antibacterial medication development. The most effective phytochemical molecules with antibacterial activity have been reported previously: pandamarilactone-1 (Hsouna et al., 2022), nonpandamarilactone-B (Hsouna et al., 2022), and thiamine (Mandal et al., 2012). The molecular docking study demonstrated that these three compounds exhibited potential inhibitory properties against the targeted bacterial protein MPh-II. Specifically, thiamine and nonpandamarilactone-B displayed more hydrogen bonding interactions with the protein, indicating a more effective drug candidate (Pagadala et al., 2013).

Indeed, the plants may provide compounds that enhance antibiotic effectiveness against resistant pathogens. These compounds may serve as resistance-modifying, modulating, or reversal agents. While standard practices often focus on screening plant extracts for direct antimicrobial properties, investigating natural resources for resistance-modifying compounds could enhance antibiotic efficacy when combined. Our study compounds have the potential for increased effectiveness and attractiveness in therapeutic applications. They align with SDG 3 (Good Health and Well-being) by investigating the possibility of *P. fascicularis* fruit extract (MEPFF) to combat antibiotic resistance, which is a significant public health threat, as they may allow the reuse of previously effective antibiotics that have lost their efficacy due to resistance. The limitation of this study is the need for *in vivo* data. Overall, more *in vivo* studies are needed to test the effect of MEPFF in combination with azithromycin. Indeed, there is a need for more studies to confirm the safety and efficacy of MEPFF *in vivo* before employing it for therapeutic purposes.

## 4 Materials and methods

### 4.1 Collection plant, extraction, and phytochemical analysis

For this investigation, 5 kg of *Pandanus fascicularis* ripened fruits of multiple sizes (37 in quantity) were obtained in May 2023 from the Sundarban region of Bangladesh. The gathered plant parts were identified by experts at the Bangladesh National Herbarium in Dhaka, where a voucher specimen was stored (voucher number MR879). The plant materials were washed with distilled water and then sun-dried for a week before being ground into a coarse powder resulting in 2786 g (55.72%) of weight. The powder was kept in a sealed glass vial and stored in a dark, cool, and dry place. Approximately 500 grams of powdered plant material were placed in a clean glass bottle and immersed in 1500 ml of methanol. The bottle was sealed and allowed to remain for a week, with periodic shaking and stirring. The mixture was then filtered using white cotton and Whatman filter paper (Bibby RE200, Sterilin Ltd., UK). The filtrate was evaporated with a rotary evaporator at 50°C. The resulting gummy concentrate was referred to as MEPFF. An appropriate amount of the extract was solubilized in methanol for phytochemical analysis. The extract solution was examined for the presence of major secondary metabolites, including phenolic compounds, saponins, flavonoids, tannins, sterols, resins, alkaloids, cardiac glycosides, and terpenoids, using standard techniques (Agidew, 2022).

### 4.2 Antioxidant activity and total antioxidant capacity

The antioxidant activity and total antioxidant capacity (TAC) of MEPFF were assessed using two distinct methods to evaluate its ability to neutralize free radicals and its overall antioxidant content. The antioxidant activity was determined using the DPPH (2,2-diphenyl-1-picrylhydrazyl) radical scavenging assay (Ashraf et al., 2015; Rashid et al., 2018; Senguttuvan et al., 2014), which measures the extract’s ability to scavenge free radicals. In detail, 3.0 ml of the methanol solution of DPPH was mixed with various concentrations of the extract. Then, the antioxidant capacity was measured by the ability of the extract to bleach a purple-colored DPPH methanol solution by determining the absorbance using a UV spectrophotometer (Shimadzu, Japan) at 517 nm. As a positive control, ascorbic acid was used. Using the following equation, the percentage of the DPPH free radical scavenging was calculated:


% of DPPH radical scavenging = [1−(As/Ac)] × 100

where Ac = absorbance of control, As = absorbance of sample solution

Then, % of DPPH scavenging was plotted against the different concentrations utilized, and the IC_50_ (the concentration of the sample required to scavenge 50% of DPPH) was determined from the straight line (linear regression) by Excel-2013 software. A lower IC_50_ and vice versa imply the higher scavenging power of the radical by the extract. This assay was specific for evaluating the radical scavenging ability of the extract against a stable free radical (DPPH), which reflected the extract’s capacity to reduce oxidative stress by neutralizing free radicals.

In contrast, the total antioxidant capacity (TAC) was measured using the phosphomolybdenum method, which evaluates the overall antioxidant content based on its ability to reduce molybdenum (VI) to molybdenum (V). In this method, 0.5 ml of extract solution (200 µg/ml) and ascorbic acid solutions (0, 10, 20,40, 80, 100 µg/ml) were added to 4.5 ml of reagent solution (0.6 M sulfuric acid, 28 mM sodium phosphate and 4 mM ammonium molybdate). The reaction solutions were incubated in a water bath at 95°C for 90 min. Then, the absorbance was measured at 695 nm using a UV spectrophotometer (Shimadzu, Japan) against blank after cooling to room temperature. From the regression equation of ascorbic acid, the concentration of ascorbic acid in the extract was determined using its absorbance. Finally, the TAC of MEPFF was calculated as milligrams of ascorbic acid equivalent per gram (mg AAE/g) of dry extract using the following equation:


$$C=C_{1}\times V/m$$

where C is TAC in mg/g in AAE (ascorbic acid equivalent), C_1_ is the concentration of ascorbic acid established from the calibration curve in mg/ml, V is the volume of the extract in ml, and m is the weight of the dry plant extract in gm (Rashid et al., 2017)TAC represents the extract’s total antioxidant content and reflects its overall ability to neutralize various reactive oxygen species. The main difference between these two methods was their focus: the DPPH assay primarily measured the extract’s ability to scavenge specific free radicals (DPPH), offering a snapshot of its radical scavenging potential. In contrast, the TAC method assessed the total antioxidant capacity of the extract, encompassing a broader range of antioxidants.

### 4.3 Total phenolic content

The Folin-Ciocalteu method was utilized to assess the total phenolic contents (TPC) of MEPFF, with slight adjustments made (Aliyu et al., 2013). To do this, 0.5 ml (200 µg/ml) of the sample was combined with 2.5 ml of 10% (v/v) Folin-Ciocalteu reagent and 2 ml of 7.5% (w/v) Na2CO3 solution. This mixture was then stored in the dark for 30 minutes at room temperature. Subsequently, absorbance was measured using a spectrophotometer (Shimadzu, Japan) at 765 nm. A standard curve was created for various concentrations (0, 10, 20, 40, 80, 100 µg/ml) of gallic acid, allowing for the determination of gallic acid concentration in the extract via the regression equation. Ultimately, the TPC in MEPFF was reported as milligrams of gallic acid equivalent per gram (mg GAE/g) of dry extract.

### 4.4 Total flavonoid content

The total flavonoid contents (TFC) of the extract were measured using the method described by Aryal et al. (2019). An aliquot of 0.5 ml of the extract solution (200 µg/ml) or quercetin (0, 10, 20, 40, 80, 100 µg/ml) was mixed with 0.2 ml of 10% (w/v) AlCl3 solution in methanol, 0.2 ml of 1 M potassium acetate, and 4.1 ml of methanol. The mixture was incubated for 30 minutes at room temperature, followed by measuring the absorbance at 415 nm against the blank. From the regression equation of quercetin, the concentration of quercetin in the extract was determined. The resulting data were expressed as mg/g of quercetin equivalents in milligrams per gram (mg QE/g) of dry extract.

### 4.5 Microorganisms and media

The study used four types of bacteria: *Staphylococcus aureus*, *Bacillus cereus*, *Escherichia coli*, and *Pseudomonas aeruginosa*. These bacteria were collected from clinical samples at the icddr,b (International Centre for Diarrheal Disease Research) in Bangladesh. All the experiments in this study were approved by the Ethical Review Committee of Varendra University (Ref: VU/ERC/2023/006). The strains of these bacteria were found to be resistant to several antibiotics, which was confirmed by testing their sensitivity to antibiotics at icddr,b. To prepare the bacteria for the study, they were grown on nutrient agar, a special medium that supports bacterial growth, and incubated at 37°C (a temperature similar to the human body) for 18 hours.

### 4.6 Antibacterial activity test by disc diffusion method

We explored the antibacterial activity of the methanol crude extract using the disc diffusion method. To prepare the test samples, we carefully dissolved specific amounts in measured volumes of DMSO. Next, we took sterilized filter paper discs, each 5mm in diameter, and gently impregnated them with 10 μl of the test substance using a micropipette before allowing them to dry in a clean aseptic hood. Then, we placed the discs containing the test material on nutrient agar medium (Merck) that was uniformly seeded with pathogenic microorganisms. Our inoculum size was approximately 10^6 cfu/ml. For this study, we utilized MEPFF discs (400 μg/disc), antibiotic azithromycin discs (10 μg/disc), and combined discs (MEPFF (200 µg/disc) + Azithromycin (5 µg/disc)). To ensure accuracy, blank discs impregnated with solvents acted as negative controls. We kept the plates at 4°C for 1 hour to allow the test material to diffuse, then incubated them at 37°C for 24 hours to promote organism growth. Any test materials that showed antibacterial activity successfully inhibited microorganism growth, leading to the formation of a clear, distinct zone of inhibition around the discs. We assessed the antibacterial activity by measuring the diameter of this zone of inhibition in millimeters.

### 4.7 Determination of minimum inhibitory concentration by broth dilution method

The MIC of MEPFF was determined through serial dilution, as described by Mogana et al. (2020). Stock solutions of the respective plant extracts were prepared in 1.5 ml microcentrifuge tubes (Eppendorf) by dissolving the dry plant extract in DMSO to achieve a final concentration of 64 mg/ml. The serial dilutions from the stock solution ranged from 32 mg/ml to 0.25 mg/ml using Mueller–Hinton broth (Becton Dickinson, Sparks, MD, USA) in 96-well microplates. A bacterial suspension containing approximately 5×10^5 colony-forming units/ml was prepared from a 24-hour culture plate. From this suspension, 100 μl was inoculated into each well, and both a sterility control well and a growth control well were analyzed for each strain. The microtiter plates were incubated at 37°C for 24 h. After incubation at 37°C, 40 μl of a 0.4 mg/ml solution of INT was added to each well to indicate microbial growth. The plates were incubated at 37°C for 30 min, and the MIC values were determined visually. The lowest concentration of each extract displaying no visible growth was recorded as the MIC. MIC values were determined in triplicate. A negative control experiment was conducted using only DMSO.

### 4.8 Determination of minimum bactericidal concentration by broth dilution method

MBC was considered as the lowest extract concentration to kill 99.9% of the bacteria after incubation at 37°C for 24 hr. The MBC was determined according to the method described by Mogana et al. (2020). Ten microliters were taken from the well of the MIC experiment (MIC value) and from two wells above the MIC value well and spread on MHA plates. After incubation for 18 to 24 hours at 37°C, the number of colonies was counted. The MBC value was defined as the concentration that resulted in fewer than 10 colonies. At least three replications of each experiment were performed.

### 4.9 Determination of fractional inhibitory concentration index

The fractional inhibitory concentration index (FICI) is employed to assess the synergism activity by the broth dilution analysis. The following formula can be used to estimate the FICI of the antibiotic-plant extract combination:


$${\text{FIC}}_{\text{plant extract}}=\frac{\text{MIC of plant extract in combination with antibiotic}}{\text{MIC of plant extract}}$$

$${\text{FIC}}_{\text{antibiotic}}=\frac{\text{MIC of plant extract in combination with antibiotic}}{\text{MIC of antibiotic}}$$

$${\text{FICI}}_{\text{plant extract in combination with antibiotic}}{\text{= FIC}}_{\text{plant extract}}{\text{+ FIC}}_{\text{antibiotic}}$$

FIC index ≤ 0.5 will be considered as synergistic, > 0.5 but < 1 as partially synergistic, additive when = 1, indifferent when > 1 but < 4 and ≥ 4 as antagonistic (Cai et al., 2007).

### 4.10 Statistical analysis

Statistical analyses were conducted to evaluate the differences among experimental groups. All experiments were performed in triplicate, and data are expressed as mean ± standard deviation (SD). To assess the statistical significance of differences between groups, a two-way analysis of variance (ANOVA) was performed. This test was used to evaluate the interaction between the experimental variables (e.g., treatment and time) and to compare differences between groups. Following the two-way ANOVA, Bonferroni post-hoc tests were applied to correct for multiple comparisons and identify pairwise differences between groups. P-values of less than 0.05 were considered statistically significant. Statistical analyses were performed using GraphPad Prism version 8.0.2 (GraphPad Software, La Jolla, CA, USA). The statistical software provided detailed results of the ANOVA and Bonferroni tests, including the F-values, degrees of freedom, and p-values for each comparison.

#### 4.10.1 Two-way ANOVA

A two-way ANOVA is a statistical method used to assess the impact of two independent variables (factors) on a dependent variable, both individually (main effects) and in combination (interaction effect). This test allows researchers to evaluate whether changes in the dependent variable are attributed to the independent variables, both separately and in their interaction. The main effects refer to the influence of each factor individually, while the interaction effect assesses whether the combined influence of the factors differs from the sum of their individual effects. Following the two-way ANOVA, a Bonferroni post-test is typically applied to control for Type I error when multiple comparisons are made. The Bonferroni post-test adjusts the significance level by dividing the chosen alpha level (usually 0.05) by the number of comparisons, thus reducing the risk of false positives in multiple pairwise comparisons. This approach ensures that statistical conclusions drawn from comparing multiple groups are more reliable and account for the increased likelihood of error when conducting multiple tests. The results of the two-way ANOVA, along with the Bonferroni correction, provide a more nuanced understanding of how the independent variables and their interaction influence the dependent variable, with corrected p-values indicating which specific comparisons are statistically significant.

### 4.11 *In silico* analysis

#### 4.11.1 Protein selection and preparation

To maintain antimicrobial efficacy and enhance the effectiveness of macrolide-based treatments, the 3D crystal structure of the protein (PDB ID: 5IGV; Macrolide 2’-phosphotransferase type II complexed with GDP and azithromycin) was extracted from the RCSB PDB database (Berman et al., 2000). This protein comprises 302 amino acids and four ligands (azithromycin, guanosin-5’-diphosphate, calcium ion, and magnesium ion) and has a resolution value of 1.55 Å from *Escherichia coli* (Fong et al., 2017). In the protein preparation stage, water, heteroatoms, ions, and cofactors were removed, and polar hydrogen atoms were added by utilizing Discovery Studio software V19.1.0.18287 (http://www.accelrys.com). Gasteiger charges were also calculated for this target protein.

#### 4.11.2 Ligands preparation

All GC-MS phytochemicals from *Pandanus fascicularis* (Adkar and Bhaskar, 2014; TL et al., 2021) were selected for molecular docking. The chemical compounds were retrieved in 3D format (.sdf) from the PubChem database (Kim et al., 2023). Their energies were minimized and optimized using PyRx tools with the ‘Uff’ force field and conjugate gradients. Finally, the files were converted to.pdbqt format.

#### 4.11.3 Active sites identification and grid box generation

Active site identification is crucial for molecular docking as it allows precise prediction of ligand binding. Identifying these regions aids in understanding protein-ligand interactions, optimizing drug design, and enhancing therapeutic efficacy by ensuring accurate binding and functional modulation within biological systems. This study employed the Biovia Discovery Studio tool to identify our protein’s active locations. First, we analyzed the crystal structure of our protein of interest, PDB ID: 5IGV, in a complex with the tiny ligand azithromycin. Then, we opened the protein and selected the active sites option. Finally, we noted down the total active locations corresponding to that protein. Regarding grid box measurements, we deleted all the heteroatoms except ZIT404 (Azithromycin) and clicked on the define site option. After that, we selected the dropdown menu named ‘from the current selection’. Next, we generated ‘SBD_Stie_Sphere’ to provide our protein’s exact grid box location. Right-clicking on Attributes of SBD_Stie_Sphere provided the details of XYZ dimensions and radius. We noted the dimensions for molecular docking runs using PyRx bioinformatics tools.

#### 4.11.4 Molecular docking

PyRxAutoDock Vina software package is vital for molecular docking due to their ability to predict ligand-protein binding interactions efficiently (Trott and Olson, 2010). Docking was performed using this tool to precisely analyze the binding interactions between the target protein and compounds obtained from *Pandanus fascicularis*. The structure of the target protein was loaded and converted into macromolecules in the software. All the converted.pdbqt ligands were selected and opened using the Open Babel interface. The previously reported grid box size was implemented to determine the appropriate ligand binding site, and the receptor grid center was placed on the active site residue of the receptor protein. The lowest-energy docked conformation was identified as the most favorable based on the docking search results. Discovery Studio was used to analyze the protein-ligand PDB complexes and examine their interactions. The binding affinity of the ligand was calculated as a negative score (kcal/mol). The docking validation process is also done.

#### 4.11.5 *In silico* drug-likeness, ADME and toxicity prediction

SMILES (Simplified Molecular Input Line Entry System) address is required to analyze the drug-likeness, ADME and Toxicity properties of a compound. The address of a compound of interest was downloaded from the PubChem database. PreADMET (http://preadmet.bmdrc.org), admetSAR (http://lmmd.ecust.edu.cn/admetsar2/), pkCSM (https://biosig.lab.uq.edu.au/pkcsm/), and ProTox-II (http://tox.charite.de/protox_II) online databases were used for the analysis of ADME and toxicity profile.

## 5 Conclusions

Infectious diseases are the second leading cause of death worldwide, following cardiovascular diseases. Despite being less highlighted than cancer, they spread quickly and evolve, challenging health systems and underscoring the need for effective prevention and control measures. Due to the rapid development of antibiotic resistance and the emergence of new disease-causing pathogens, there is an urgent need to discover new antimicrobial drugs. MEPFF has shown strong synergistic effects with azithromycin against multi-drug-resistant gram-positive and gram-negative bacteria. It contains important bioactive compounds, including terpenoids, flavonoids, and alkaloids, which help combat pathogenic diseases. In silico experiments identified pandamarilactone-1, nonpandamarilactone-B, and thiamine as the most effective compounds against bacterial proteins. These compounds demonstrated significant antibacterial activity and adhered to Lipinski’s Rule of Five, suggesting their potential for further testing in animal models and clinical trials as promising antibacterial agents. While the combination of MEPFF and azithromycin shows promise, it is premature to claim its role in the development of safe and effective medications without *in vivo* and clinical data. These findings emphasize the need for additional research to validate the therapeutic potential of these compounds, laying the groundwork for future exploration of their medicinal properties.

## Data Availability

The raw data supporting the conclusions of this article will be made available by the authors, without undue reservation.
