# Supplementary material for: Synergistic effects of Pandanus fascicularis extracts and azithromycin: in vitro and in silico antimicrobial investigation against MDR clinical strains
Source: Front Antibiot. 2025 May 29;4:1552382. doi: 10.3389/frabi.2025.1552382 (PMC12159755; doi:10.3389/frabi.2025.1552382)
Supplement: Supplementary file 1 [file Table1.docx]

**Supplementary Table**

**Table S1:** List of molecular docking compounds.

| **Compound’s CID** | **Binding Affinity kcal/mol** |
| --- | --- |
| **102224960** | **-9.9** |
| **11073796** | **-8.9** |
| **1130** | **-8.5** |
| **131752721** | **-7.9** |
| **272784151** | **-7.5** |
| **447043** | **-6.9** |
| **493570** | **-6.2** |
| **54670067** | **-6** |
| **641595** | **-5.7** |
| **85389398** | **-5.2** |
| **938** | **-4.8** |
| **552019** | **-3.9** |
| **6680** | **-6.4** |
| **10560** | **-2.3** |
| **31256** | **-3.2** |
| **2969** | **-3.9** |
